# Business Intelligence System for Government and the Community

**DOI:** 10.23889/ijpds.v1i1.398

**Published:** 2017-04-19

**Authors:** Chodziwadziwa Whiteson Kabudula, Georges Reniers, Francesc Xavier Gómez-Olivé, Kathleen Kahn, Stephen Tollman

**Affiliations:** 1 London School of Hygiene and Tropical Medicine; 2 University of the Witwatersrand

## Objectives

Business Intelligence System for Government and the Community's goal is to assist the delivery of quality publically funded services and infrastructure informed by evaluation and research. Governments drive for open data can facilitate reduced budget expenditure and maximised value and benefit to citizens from government funded services and infrastructure. The challenge is to incorporate non-for-profit and for-profit organisation's data into this business intelligence and reporting capacity. Obviously there are necessary governance issues to be resolved to include non-government data. These issues include ensuring the legal release of data as well maintaining the trust of the community through adequate data security, privacy protection and demonstration of public benefit from the use of sensitive data. In building on the government's capability to bring together data for public good purposes, the wider community needs to be included for the ability to monitor, evaluate and perform research at a unit record level on publically funded government and non-for-profit provided services. 

## Approach

It is critically important to demonstrate trust to both the community and delegated Data Custodians responsible for approving data release and use. This ability to bridge across the government and community has three goals including:

1. Striving for world class public funded services and infrastructure including Health & Wellbeing, Education, Training, Economic, Employment, Criminal and Justice, Social Services.
2. Providing the evidence base to inform Policy, Planning, Evaluation, Monitoring of Programs, and to promote Innovation.
3. Providing cost effective and appropriate service delivery, infrastructure and programs - informed by Science & Research.

With multiple tiers of government and multiple agencies collecting and maintaining large databases, resulting in silos which prevent bringing together an evidence base, consideration is required on how best to collect and utilise the data from the non-for-profit and for-profit sectors to inform government planning, priorities and spending.

A federated model is required to bring together data from multiple agencies and organisations, with the critical role of a trusted third party data broker for assurance of privacy protecting and governance.

## Results

There are significant challenges from multiple jurisdictions and tiers government, and the need to facilitates legal data access for ethically approved use. Legislative reform is required to provide the approved processes to demonstrate assurance to the community, and enable data custodians participation. 

## Conclusion

A Business Intelligence System for Government and the Community requires political leadership, the establishment of trust and the active engagement across the community.

